# Effects of sacubitril/valsartan on exercise capacity: a prognostic improvement that starts during uptitration

**DOI:** 10.1007/s00228-023-03527-y

**Published:** 2023-06-27

**Authors:** Massimo Mapelli, Irene Mattavelli, Stefania Paolillo, Elisabetta Salvioni, Damiano Magrì, Arianna Galotta, Fabiana De Martino, Valentina Mantegazza, Carlo Vignati, Immacolata Esposito, Simona Dell’Aversana, Roberta Paolillo, Teresa Capovilla, Gloria Tamborini, Alessandro Alberto Nepitella, Pasquale Perrone Filardi, Piergiuseppe Agostoni

**Affiliations:** 1 Centro Cardiologico Monzino, IRCCS, Milan, Italy; 2 Department of Clinical Sciences and Community Health, Cardiovascular Section, University of Milan, Milan, Italy; 3 Department of Advanced Biomedical Sciences, Federico II University of Naples, Naples, Italy; 4 Department of Clinical and Molecular Medicine, University “La Sapienza”, Rome, Italy; 5 Casa di Cura Tortorella, Salerno, Italy; 6 University of Trieste, Trieste, Italy; 7 Policlinico Universitario D. Casula, Cardiologia – AOU Cagliari Cagliari, Italy

**Keywords:** Sacubitril/valsartan, Heart failure, Exercise capacity, Cardiopulmonary exercise test, Biomarkers

## Abstract

**Purpose:**

Sacubitril/valsartan is a mainstay of the treatment of heart failure with reduced ejection fraction (HFrEF); however, its effects on exercise performance yielded conflicting results. Aim of our study was to evaluate the impact of sacubitril/valsartan on exercise parameters and echocardiographic and biomarker changes at different drug doses.

**Methods:**

We prospectively enrolled consecutive HFrEF outpatients eligible to start sacubitril/valsartan. Patients underwent clinical assessment, cardiopulmonary exercise test (CPET), blood sampling, echocardiography, and completed the Kansas City Cardiomyopathy Questionnaire (KCCQ-12). Sacubitril/valsartan was introduced at 24/26 mg b.i.d. dose and progressively uptitrated in a standard monthly-based fashion to 97/103 mg b.i.d. or maximum tolerated dose. Study procedures were repeated at each titration visit and 6 months after reaching the maximum tolerated dose.

**Results:**

Ninety-six patients completed the study, 73 (75%) reached maximum sacubitril/valsartan dose. We observed a significant improvement in functional capacity across all study steps: oxygen intake increased, at peak exercise (from 15.6 ± 4.5 to 16.5 ± 4.9 mL/min/kg; *p* trend = 0.001), while minute ventilation/carbon dioxide production relationship reduced in patients with an abnormal value at baseline. Sacubitril/valsartan induced positive left ventricle reverse remodeling (EF from 31 ± 5 to 37 ± 8%; *p* trend < 0.001), while NT-proBNP reduced from 1179 [610–2757] to 780 [372–1344] pg/ml (*p* trend < 0.0001). NYHA functional class and the subjective perception of limitation in daily life at KCCQ-12 significantly improved. The Metabolic Exercise Cardiac Kidney Index (MECKI) score progressively improved from 4.35 [2.42–7.71] to 2.35% [1.24–4.96], *p* = 0.003.

**Conclusions:**

A holistic and progressive HF improvement was observed with sacubitril/valsartan in parallel with quality of life. Likewise, a prognostic enhancement was observed.

## Introduction

Heart failure with reduced ejection fraction (HFrEF) still represents a major issue in the general population with a continuously increasing prevalence [1] and a persistently high 5-year mortality rate, also in patients considered in “stable” clinical conditions [2, 3]. Sacubitril/valsartan therapy is a cornerstone of HFrEF pharmacological treatment due to its favorable effect on cardiovascular death and heart failure (HF) hospitalizations [4–6]. Despite this positive prognostic impact, conflicting results have emerged on the effects of sacubitril/valsartan on exercise performance with some studies showing an improvement of exercise tolerance [7–13] and some others showing no significant effects [14–18]. These studies differ in terms of numerosity, study design (randomized vs. observational), and/or methodology. Indeed, exercise performance has been differently evaluated by means of maximal symptoms limited cardiopulmonary exercise test (CPET) [7–11, 14, 15, 17, 18], 6-min walking test [12, 13, 16, 19], and using exercise surrogates such as data obtained from accelerometer devices [14, 16]. Notably, little is known about exercise performance changes during sacubitril/valsartan uptitration [7].

CPET is a valuable tool in HFrEF, allowing accurate assessment of patients’ functional capacity and providing prognostically relevant parameters (e.g., oxygen intake at peak [peakVO_2_] and at anaerobic threshold [AT], and minute ventilation/carbon dioxide production relationship [VE/VCO_2_ slope]) [20, 21].

The aim of the present study was to prospectively evaluate the effects of sacubitril/valsartan on exercise performance, cardiac remodeling, functional status, and quality of life in a large population of HFrEF patients, after drug introduction and at different drug doses.

## Materials and methods

We prospectively enrolled HFrEF outpatients referred to the HF Unit of three Italian Institutes between December 2018 and December 2019, who were eligible to start sacubitril/valsartan according to 2016 ESC Guidelines [22]. Study inclusion criteria were age > 18 years, males and females, New York Heart Association (NYHA) Class II–III in stable clinical condition, left ventricular ejection fraction (LVEF) ≤ 35%, and ability to perform CPET. Patients affected by chronic obstructive pulmonary disease or in need of oxygen supplement were excluded.

At baseline, each patient underwent all study procedures while taking their background guideline-directed therapy for HF at the maximum tolerated dose. After 36 h of interruption of angiotensin-converting enzyme inhibitors (ACE-I) or angiotensin receptor blockers (ARBs), sacubitril/valsartan was introduced at 24/26 mg b.i.d. starting dose for all patients. After enrollment, the drug was progressively uptitrated in a standard monthly-based fashion to 97/103 b.i.d. or to the maximum tolerated dose.

**Fig. 1:**
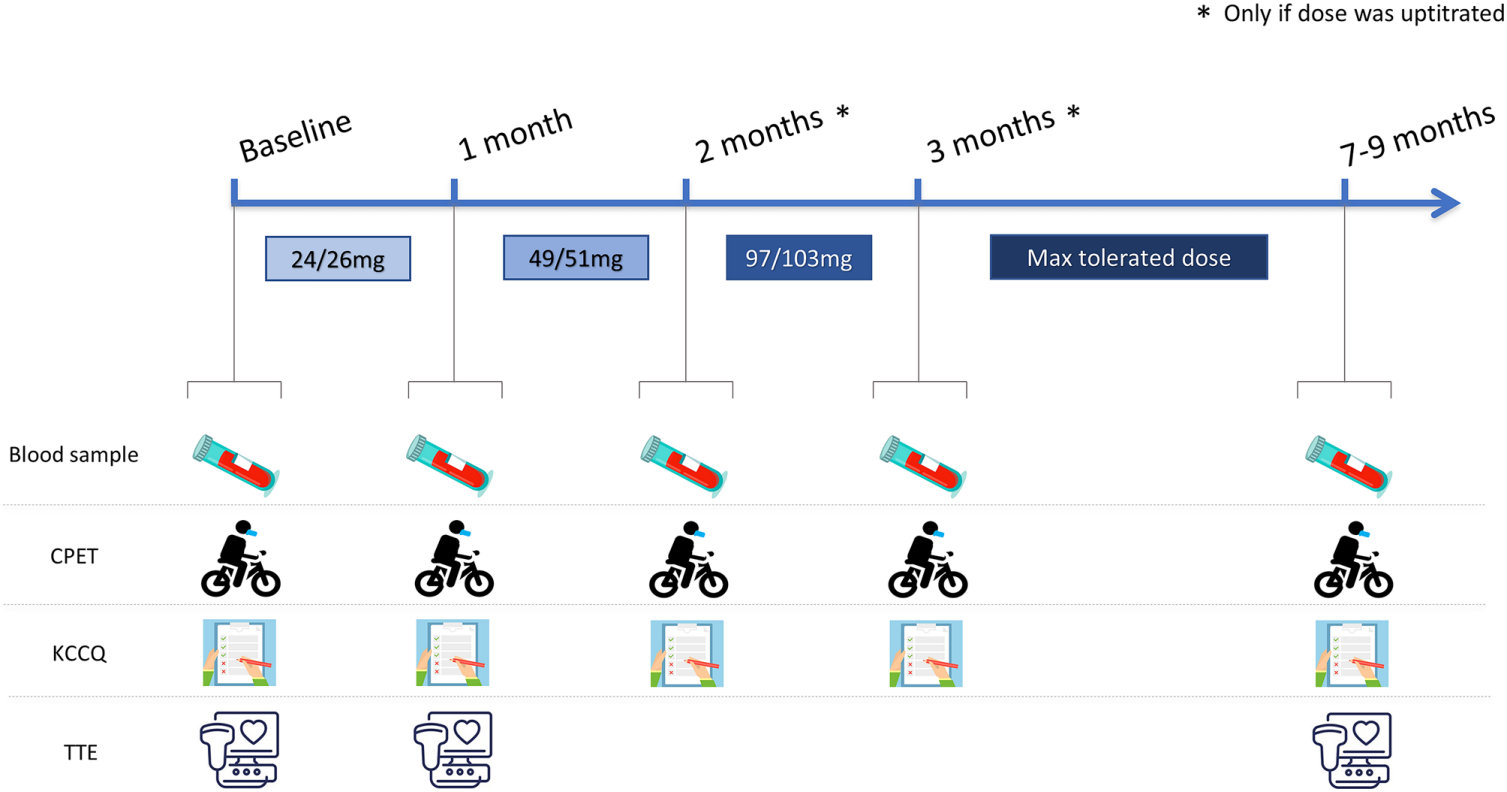
Study protocol. The figure shows the study procedures performed at each study-step, during the progressive monthly-based drug uptitration and at the end of the study. Specifically, each patient underwent at each step a blood sample (renal function assessed by the MDRD equation, serum potassium and sodium, NT-proBNP, Hb), a maximal CPET with the same ramp protocol, and a QoL evaluation through KCCQ-12. TTE was performed at baseline, after the first month, and at the end of the study. Abbreviations: MDRD, Modification of Diet in Renal Disease; NT-proBNP, N-terminal pro B-type natriuretic peptide; Hb, hemoglobin; CPET, cardiopulmonary exercise test; QoL, quality of life; KCCQ-12, Kansas City Cardiomyopathy Questionnaire, TTE, transthoracic echocardiography

All study procedures were performed at baseline, repeated at each titration visit and 6 months after the maximum tolerated dose was reached (except for echocardiography that was repeated only after 1 month and at the end of the study). Specifically, patients underwent clinical assessment, CPET, venous blood sample collection, and transthoracic echocardiography (TTE). Moreover, quality of life (QoL) was evaluated through Kansas City Cardiomyopathy Questionnaire (KCCQ-12) at each study visit, administrated before any other assessment (Fig. 1).

### Cardiopulmonary exercise testing

In all laboratories patients were tested by a stationary ergospirometer (Quark PFT Cosmed, Rome, Italy) using an electronically braked cycle ergometer and wearing a standard CPET silicone mask, using a ramp protocol set to achieve peak exercise in ~ 10 min [23]. To allow comparison among CPET results, the ramp protocol was chosen for each patient at the baseline visit and then applied to all the subsequent tests. In the absence of clinical events, the exercise was interrupted when patients stated that they had reached maximal effort. We performed a breath-by-breath analysis of expiratory gases and ventilation. The VO_2_/work rate relationship was measured throughout the entire exercise. The AT was identified using a V-slope analysis of VO_2_ and CO_2_ production (VCO_2_), and it was confirmed by specific trends of ventilation vs VO_2_ and ventilation vs CO_2_ and of end-tidal pressure of oxygen and end-tidal pressure of CO_2_. AT was reported as absolute value and as percentage of the predicted maximum VO_2_ [24, 25]. The VE/VCO_2_ slope was calculated from 1 min after the beginning of the loaded exercise to the end of the isocapnic buffering period. Predicted values of peak VO_2_ were calculated as “(height − age) × 20” for men and “(height − age) × 14” for women, with height expressed in centimeters and age in years [26].

### Transthoracic echocardiography

TTE examinations were performed using Philips ultrasound machine (Epiq CVx – Philips Medical Systems, Andover, Massachusetts) equipped with an X5-1 probe. Complete standard 2D TTE analysis was performed. Left chambers’ volumes and LVEF were measured from 4-chamber and 2-chamber views using the biplane Simpson’s method [27]. All echocardiograms were performed by well-trained operators.

### Kansas city cardiomyopathy questionnaire analysis

The KCCQ-12 was analyzed combining the reported Physical Limitation, Symptom Frequency, Quality of Life and Social Limitation scales into the Summary Score, calculated as the average of the available scale scores. In order to calculate the summary score, at least one of the four scale scores must be present [28, 29].

### Prognostic score

As additive analysis, we included a multiparametric prognostic risk assessment through Metabolic Exercise Cardiac Kidney Index (MECKI) score calculation, to analyze the impact of sacubitril/valsartan introduction on two-year risk of death or urgent heart transplant/LVAD (left ventricle assist device) implantation. The MECKI score was calculated as previously described [30].

### Statistical analysis

Statistical analyses were performed using SAS software, version 9.4 (SAS Institute, Cary, NC, USA). Continuous variables were expressed as means ± standard deviation (SD) or median and interquartile range [IQR] in case of non-normally distributed variables; categorical variables were reported as frequencies and percentages. Missing values (in V1, V2, and V3) were imputed by considering the value of the following visit, where present. Comparisons of observed values over time were performed by repeated-measures ANOVA, after log-transformation of variables with right-skewed distribution; single comparisons were performed by paired *t*-tests and Bonferroni’s correction for multiple comparisons was applied. Pearson’s or Spearman’s correlation coefficient was calculated, as appropriate. All tests were two-sided, and a *p* value < 0.05 was required for statistical significance (after Bonferroni’s correction where appropriate).

The present research protocol complies with World Medical Association Declaration of Helsinki, and it was approved by the Centro Cardiologico Monzino Ethical Committee (CCM 898) as the guiding study center as well as by the local Ethical Committee of satellites centers. Each subject provided written informed consent to the study. This observational cohort study was also registered into clinicaltrials.gov on June 16, 2020, with ID: NCT04434170.

## Results

One hundred and thirteen HFrEF outpatients (81% males, age 64.5 ± 9.7 years) were enrolled in three Italian centers. Seventeen patients (15%) interrupted the protocol for the following reasons: 1 patient experienced sudden cardiac death; 5 patients had clinical worsening (1 renal function worsening and 4 symptomatic hypotension) and interrupted the study drug; 2 patients were diagnosed with cancer; 2 patients were excluded after unscheduled cardiac resynchronization therapy (CRT) implantation; 1 patient had SARS-CoV-2 infection with respiratory failure, for 4 patients, CPET was contraindicated due to ventricular arrhythmias/mobile left ventricular thrombosis; and 2 patients withdrew from the study for personal reasons. All these patients were excluded from the analysis.

Table 1 shows the main characteristics of the retained population and therapy at enrolment (*n* = 96). HFrEF was of ischemic etiology in 55% of patients, 59 patients (59%) had hypertension, and 38 (39%) chronic kidney disease with eGFR ≤ 60 ml/min/1.73m^2^. At a mean follow-up of 282 ± 63 days, 72 patients (75%) reached the maximum sacubitril/valsartan dose (97/103 mg b.i.d.) without safety concerns. After the baseline assessment, a further functional evaluation was not possible in 4 patients due to physical limitations and/or poor compliance/motivation to the test.

**Table 1:** Basal characteristics of the retained study population (*n* = 96)

	*Data at enrolment*
***Age (years)***	63.66 ± 9.80
***BMI (kg/m***^***2***^***)***	27.08 ± 4.24
***SBP (mmHg)***	117.60 ± 15.49
***Heart rate (bpm)***	66.92 ± 10.73
***Males (n, %)***	77, 80%
***Ischemic etiology (n, %)***	53, 55%
***Hypertension (n, %)***	57, 59%
***Diabetes (n, %)***	21, 22%
***Atrial fibrillation (n, %)***	23, 24%
***ICD (n, %)***	36, 38%
***CRT-D (n, %)***	21, 22%
***NYHA II (n, %)***	84, 88%
***NYHA III (n, %)***	11, 11%
***Hemoglobin (g/dL)***	14.04 ± 1.60
***Creatinine (mg/dL)***	1.16 ± 0.26
***GFR (ml/min/1,73 m***^***2***^***)***	68.92 ± 19.58
***Sodium (mmol/L)***	140.72 ± 2.79
***Potassium (mmol/L)***	4.40 ± 0.42
***ACE-I (n, %)***	72, 75%
***ARB (n, %)***	22, 23%
***BB (n, %)***	95, 99%
***MRA (n, %)***	69, 72%
***Loop diuretics (n, %)***	76, 79%

*BMI* body mass index, *SBP* systolic blood pressure, *ICD* implantable cardioverter defibrillator, *CRT-D* cardiac resynchronization therapy-defibrillator, *NYHA* New York Heart Association, *GFR* glomerular filtration rate assessed by Modification of Diet in Renal Disease (MDRD) equation, *ACE-I* angiotensin-converting-enzyme inhibitor, *ARB* angiotensin receptor blocker, *BB* beta blocker, *MRA* mineralocorticoid receptor antagonist

**Fig. 2:**
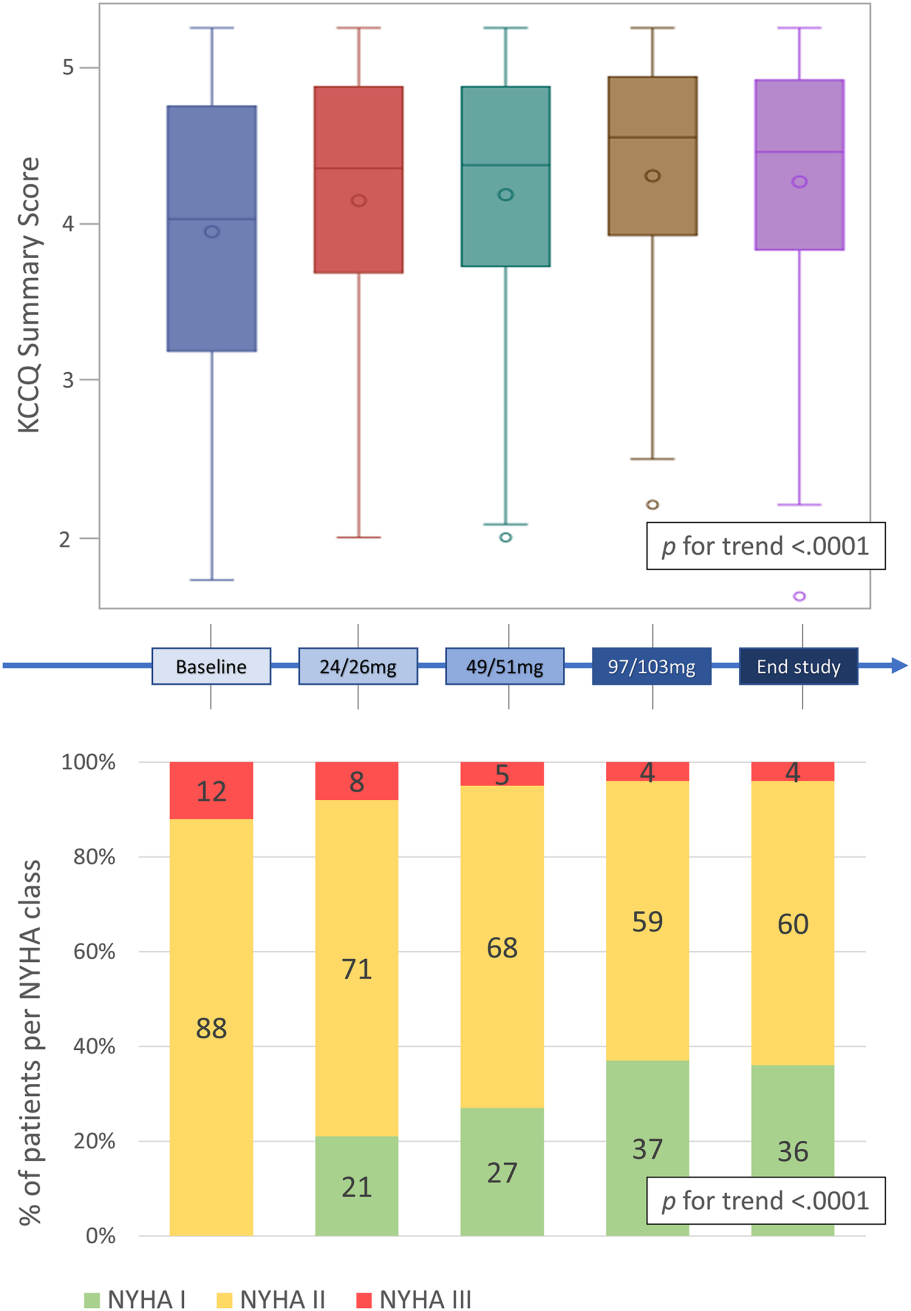
Quality of life and NYHA class amelioration during the study. A progressive significant improvement in QoL (upper panel) and NYHA class (lower panel) was observed during the drug uptitration. KCCQ-12 summary score significantly improved from 3.95 ± 0.92 to 4.27 ± 0.81. About one-fifth of patients report being asymptomatic (NYHA I) already after 1 month of treatment at the lower sacubitril/valsartan dose (24/26 mg b.i.d.), and more than one-third become asymptomatic at the end of the study. Abbreviations: QoL, quality of life; NYHA, New York Heart Association; KCCQ, Kansas City Cardiomyopathy Questionnaire

Compared to baseline (Table 1), NYHA functional class significantly improved at the end of study assessment with 34 (36%) patients in class 1, 57 (60%) in class 2 and only 4 (4%) in class 3 (*p* < 0.001) (Fig. 2). From the analysis of KCCQ-12 summary score, we observed a statistically significant improvement of the subjective perception of functional limitation in daily life, from a score of 3.95 ± 0.92 at baseline to 4.27 ± 0.81 at end of study (Fig. 2).

**Fig. 3:**
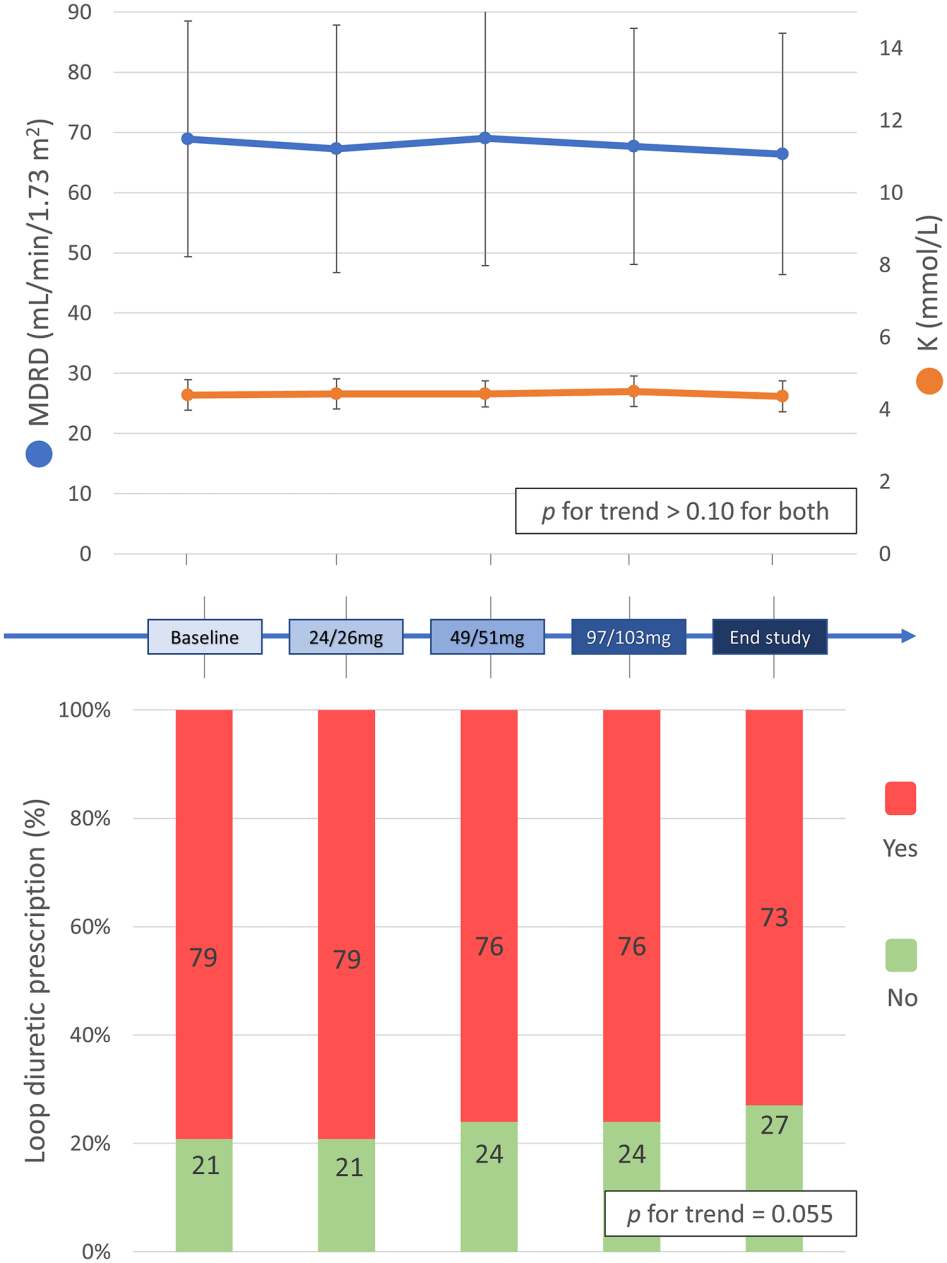
Renal function, potassium values and loop diuretic prescription. The upper panels show how renal function (as assessed by MDRD) and serum potassium did not significantly change during the whole study. In the lower panel, a progressive, non-significant, reduction in the prescription of loop diuretics (e.g., furosemide) is shown. Abbreviations: MDRD, Modification of Diet in Renal Disease

As regards safety concerns, we did not observe a significant change in renal function and electrolytes (eGFR from 69 ± 20 to 66 ± 20 ml/min/1.73m^2^, potassium from 4.40 ± 0.42 to 4.36 ± 0.43 mmol/l, *p* = ns for both) (Fig. 3A), while a statistically significant — however not clinically relevant — reduction in systolic blood pressure was observed during sacubitril/valsartan uptitration (from 118 ± 16 to 109 ± 14 mmHg, *p* for trend < 0.001). We recorded an almost significant reduction in the prescription of loop diuretics during the whole study period (Fig. 3B).

**Fig. 4:**
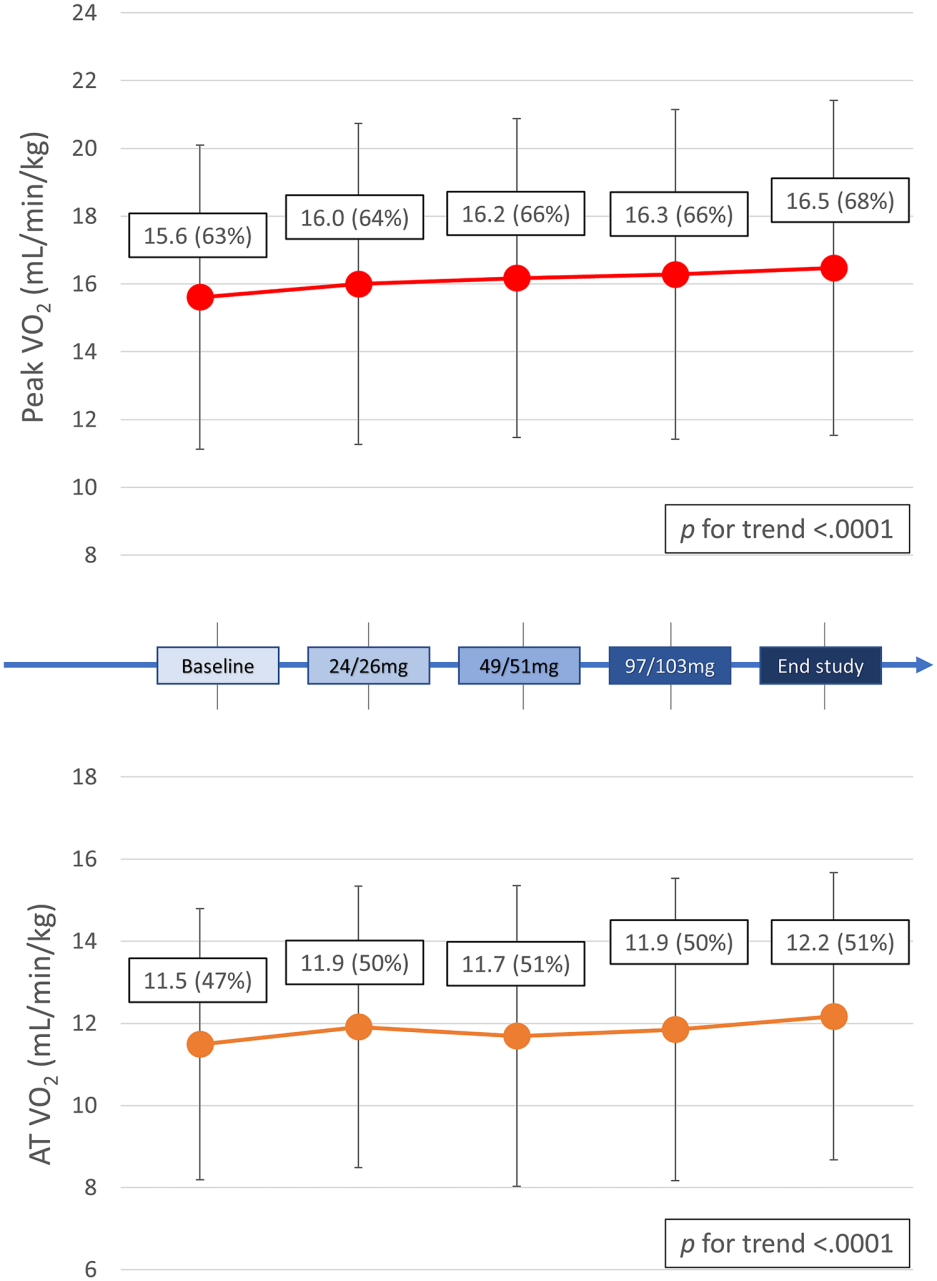
Oxygen intake progressive improvement. The figure shows the progressive significant improvement of VO_2_, both at peak exercise (upper part) and at the AT (lower part). As demonstrated by the p for trend values, the increase in VO_2_ starts during the first uptitration steps and is maintained through the whole study protocol. Abbreviations: VO_2_, oxygen intake; AT, anaerobic threshold

On the average, we observed a significant improvement in functional capacity parameters. From baseline to the end of the study, peak VO_2_ increased from 15.6 ± 4.5 to 16.5 ± 4.9 mL/min/kg (*p* = 0.001) with a progressive trend of improvement throughout all titration visits (*p* for trend = 0.001) (Fig. 4, Table 2). Notably, peak VO_2_ changes were in parallel with NYHA class amelioration (Table 3). VO_2_ at the AT, detectable in 80 patients, significantly improved, from 11.5 ± 3.3 ml/min/kg (47 ± 14% of the predicted peak VO_2_) to 12.2 ± 3.5 mL/min/kg (51 ± 15%) (*p* value baseline vs. end of study 0.028) (Fig. 4, Table 2). Peak workload increased as well across all the study steps (form 91 [67–120] to 97 [73–121] Watt, *p* for trend < 0.001). We did not observe a significant reduction in VE/VCO_2_ slope values in the whole population (Table 2). Differently, considering only the frailer patients with pathological slope value at baseline (VE/VCO_2_ ≥ 34, *n* = 34), a significant amelioration was observed at the end of the study, from 39.5 ± 5.8 to 35.9 ± 7.1 (*p* for trend = 0.030).

**Table 2:** Cardiopulmonary exercise test and echocardiographic parameters during the study

	***Baseline***	***24/26 mg***	***49/51 mg***	***97/103 mg***	***End study***	***p TREND***
***Peak HR (bpm)***	109 ± 24	108 ± 23	107 ± 23	109 ± 23	111 ± 23	0.334
***Peak HR (% pred)***	70.3 ± 15.3	70.1 ± 15.1	68.8 ± 14.3	71.0 ± 16.3	71.1 ± 13.5	0.429
***Peak power (watt)***	91 (67–120)	98 (72–123)	99 (68–125)	101 (71–122)	97 (73–121)	0.001
***VO***_***2***_*** AT (mL/min/kg)***	11.5 ± 3.3	11.9 ± 3.4	11.7 ± 3.7	11.9 ± 3.7	12.2 ± 3.5	0.245
***VO***_***2***_*** AT (% pred)***	47 ± 14	50 ± 17	51 ± 18	50 ± 15	51 ± 15	0.085
***Peak VO***_***2***_*** (mL/min/kg)***	15.6 ± 4.5	16.0 ± 4.7	16.2 ± 4.7	16.3 ± 4.9	16.5 ± 4.9	0.001
***Peak VO***_***2***_*** (% pred)***	63 ± 16	64 ± 17	66 ± 16	66 ± 17	68 ± 17	< 0.0001
***VE/VCO***_***2***_*** slope***	32.8 ± 6.8	31.6 ± 6.3	32.1 ± 6.3	31.8 ± 6.5	32.1 ± 6.0	0.952
***O***_***2***_*** pulse (mL/b)***	11.9 ± 4.1	12.1 ± 3.4	12.4 ± 3.7	12.3 ± 3.8	12.1 ± 3.5	0.015
***LAVi (mL/m***^***2***^***)***	44.3 (34.8–56.8)	39.6 (34.0–51.2)			37.9 (33.0–51.3)	< 0.0001
***MR grade***	1 (1–2)	1 (1–1.5)			1 (1–1)	< 0.0001
***LVEDVi (mL/m***^***2***^***)***	94 (79–114)	90 (73–107)			84 (67–98)	< 0.0001
***LVESVi (mL/m***^***2***^***)***	63 (54–80)	58 (45–72)			53 (39–68)	< 0.0001
***LVEF (%)***	31.4 ± 4.7	34.3 ± 7.3			36.6 ± 8.1	< 0.0001
***TAPSE (mm)***	20.4 ± 4.5	19.6 ± 4.3			19.8 ± 4.7	0.081
***PAPS (mmHg)***	33.7 ± 11.8	31.2 ± 15.1			29.9 ± 13.7	0.007
***MECKI score (%)***	4.35 (2.42–7.71)	2.95 (1.92–6.74)			2.35 (1.24–4.96)	< 0.0001

*HR* heart rate, *VO2* oxygen intake, *AT* anaerobic threshold, *VE/VCO2* minute ventilation/carbon dioxide production relationship, *LAVi* left atrial volume indexed, *MR* mitral regurgitation, *LVEDVi* left ventricle end diastolic volume indexed, *LVESVi* left ventricle end systolic volume indexed, *LVEF* left ventricle ejection fraction, *TAPSE* tricuspid annular plane systolic excursion, *PAPS* systolic pulmonary artery pressure, *MECKI* Metabolic Exercise Cardiac Kidney Index

**Table 3:**
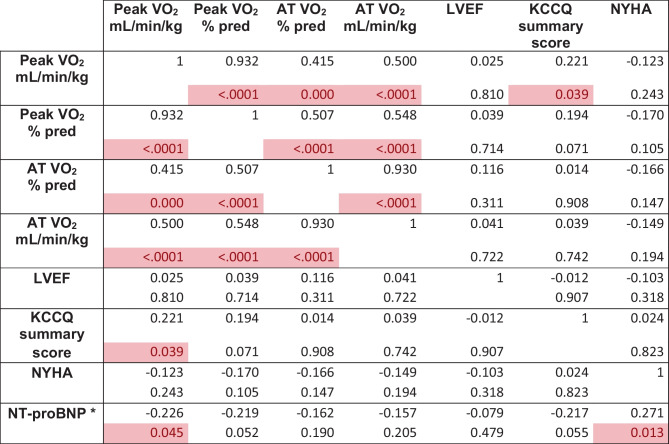
Correlations

Significant (p < 0.05) correlations are highlighted in red

*VO2* oxygen intake, *AT* anaerobic threshold, *LVEF* left ventricle ejection fraction, *KCCQ* Kansas City Cardiomyopathy Questionnaire, *NYHA* New York Heart Association Class

*Spearman correlation

In parallel, sacubitril/valsartan induced a positive cardiac reverse remodeling (Table 2). Specifically, LVEF increased (31 ± 5 vs. 37 ± 8%; *p* for trend < 0.001); end-diastolic and end-systolic volumes (EDV and ESV) decreased [from 194 ± 66 to 170 ± 60 ml (*p* for trend < 0.001) and from 122 [104–160] to 101 [77–141] ml (*p* for trend < 0.001), respectively] along with systolic pulmonary artery pressure (PAPS) (from 34 ± 12 to 30 ± 14 mmHg, *p* = 0.007). We observed a significant reduction in left atrial (LA) volume from 44.3 [34.8–56.8] to 37.9 [33.0–51.3] mL/m^2^, *p* for trend < 0.0001. Of note, changes in peak VO_2_ and LVEF were independent of each other (Table 3).

As regards cardiac biomarkers, NT-proBNP significantly reduced across the study steps from 1179 [610–2757] to 780 [372–1344] pg/ml (*p* for trend < 0.0001) (Table 2). Of note, we observed a statistically significant reduction (*p* < 0.0001) at all study steps compared to baseline values. Again, NT-proBNP amelioration was independent from LVEF improvement, but was in parallel with peak VO_2_ and NYHA class changes (Table 3).

Finally, a global, multiparametric risk assessment was evaluated through MECKI score. We observed a statistically significant reduction from baseline of the calculated risk at 2 years, from 4.35 [2.42–7.71] to 2.35% [1.24–4.96], *p* = 0.003 (Table 2).

## Discussion

The present study demonstrated a positive impact of sacubitril/valsartan on exercise capacity, reverse cardiac remodeling, NT-proBNP, and QoL in stable HFrEF patients. Importantly, for the first time, we showed how these improvements, although not directly correlated with each other from a statistical point of view, are consistent across the whole fixed monthly-based uptitration steps with the commercially available drug dosages (24/26 ➔ 49/51 ➔ 97/103 mg).

In particular, we evidenced an improvement of the strongest prognostically relevant CPET parameters, as shown by a significant increase in peak VO_2_, together with a reduction in VE/VCO_2_ slope in frailer patients. Moreover, a tendency toward VO_2_ improvement at AT was observed (*p* for trend ns; *p* value baseline vs end study 0.028). This result is in line with the increased number of patients in which AT became detectable during the treatment (from 87 to 95% at the end of the study). This is particularly relevant being the presence of a non-detectable AT linked to a non-homogenous oxygen delivery to the working muscles to non-homogenous resistance to oxygen flow from the capillary to the mitochondria and muscle oxygen utilization. Moreover, the presence of a non-detectable AT is per se a well-known strong negative prognostic marker in HFrEF [25, 31].

These data should be read in relation to previous studies on this topic. In the ACTIVITY-HF study [14], a randomized controlled trial assessing functional capacity with CPET, sacubitril/valsartan failed to demonstrate a benefit on peak VO_2_ compared with enalapril on a large multicenter population of HFrEF patients. Another randomized trial by Dos Santos et al. [15] reached the same conclusions, as another small Italian retrospective trial did [18]. One possible explanation is that the populations enrolled in these studies are different from ours, as evidenced by baseline peak VO_2_ values, particularly low in ACTIVITY-HF (12.9 ± 3.0 ml/kg/min) and high in the Dos Santos et al. study (19.35 ± 0.99 ml/kg/min). It is likely that, while maintaining a favorable prognostic effect, in patients who are too advanced or too “healthy,” sacubitril/valsartan may not be able to improve functional capacity, as our data seem to suggest this effect in an intermediate “sick” HF population (baseline peak VO_2_ 15.6 ± 4.5 ml/kg/min). In a comparable group of HFrEF patients (baseline peak VO_2_ 14.6 ± 3.3 ml/Kg/min) enrolled in a single arm prospective study, Vitale et al. [7] demonstrated an increase of 17.8% in peak VO_2_ at 6.2-month follow-up and an improvement in other CPET parameters, as evidenced by a significant reduction in VE/VCO_2_ slope values in frailer patients. On the other hand, a previous small study conducted on a selected population of advanced HFrEF patients listed for heart transplantation seems to suggest a residual favorable effect of sacubitril/valsartan on exercise capacity [32]. All these data indicate that accurately predicting which patients are more likely to show an improvement in functional capacity during sacubitril/valsartan therapy is not straightforward. The treatment response is indeed variable also depending on phenotypic features of the disease underlying HFrEF, as evidenced in a trial by Nugara et al., in which functional benefit correlated with the presence of myocardial fibrosis on cardiac magnetic resonance [33]. Therefore, the presence of a “sweet spot” in baseline VO_2_ as a possible predictor for maximal benefit during sacubitril/valsartan treatment represents to date a fascinating hypothesis but requires further study to be definitely demonstrated.

Importantly, our trial was the first one designed to assess a dose/effect relationship with a fixed and progressive monthly-based uptitration of the drug dosages. We demonstrated how the more relevant CPET parameters, as well as NT-proBNP, NYHA class and QoL, start to improve since the first step (24/26 mg b.i.d.), maintaining a progressive and sustained improvement during the entire follow-up. This suggests that also low doses of sacubitril/valsartan may already be important in conferring clinical and prognostic benefits, as also shown by an observational study by Corrado et al. [34].

The CPET changes were paralleled by a decrease of LV volumes (both EDV and ESV), LA volumes, PAPs and MR, as previously published by our group [35, 36]. Interestingly, we showed that 32 patients (33%) improved LVEF from < 40 to more than 40%, meeting the criteria for HF with improved EF (HFimpEF). This subset of HF patients is now properly classified as a new group by the HF American guidelines [37], with the aim to underline the prognostic relevance of a dynamic evaluation of the HF patients. Similarly, 42 patients (51%) improved EF from < 35 to more than 35%, losing the formal indication for ICD implantation in primary prevention [38], and confirming the results from a recently published article [39].

Parallel to the functional capacity improvement at CPET, we also observed an amelioration in patients’ subjective perception of functional limitation during their daily life, assessed through NYHA class and, importantly, with the KCCQ-12 questionnaire (Fig. 3), a well-validated prognostic tool in the HFrEF field [40]. Interestingly, after the baseline evaluation in which only NYHA II and III HFrEF patients were enrolled, about one-fifth of patients reported being asymptomatic (NYHA I) already after 1 month of treatment at the lower sacubitril/valsartan dose (24/26 mg b.i.d.), and more than one-third (36%) become asymptomatic by the end of the study (Fig. 3). These results are in line with a significant increase in peak workload at CPET, confirming that patients are able to tolerate more intense efforts with ongoing sacubitril/valsartan treatment. Even in the context of a general improvement of most of the variables evaluated, the significant correlation of VO_2_ with KCCQ changes and not with an echocardiographic variable such as LVEF is interesting, thus showing how the evaluation of functional capacity represents a fundamental, holistic approach which better represents the patient’s state and wellness, beyond its prognostic aspect.

Looking into the need for a more dynamic evaluation of HFrEF (each patient’s participation in our trial lasted about 9 months), all these changes are particularly relevant since they occurred “against the current,” whereas the natural evolution of this disease would have been a progressive worsening. Moreover, while the standard background HF therapy was kept stable during the observation period, we noted a reduction in the prescription of furosemide (Fig. 3), showing how the observed favorable effects are independent from loop diuretic administration and actually allow a diuretic dose reduction which is considered a positive trend due to the diuretic drawbacks. Once again, sacubitril/valsartan safety and the tolerability were confirmed by stable renal function and potassium values over time, and by 75% of the patients being able to tolerate the 97/103 mg b.i.d dose without adverse reactions.

The positive prognostic effects of sacubitril/valsartan were also confirmed by the risk assessment provided by the combination of metabolic, exercise, cardiac, and kidney function parameters into the MECKI score [30]. Specifically, the risk of death or urgent heart transplant/LVAD implantation at 2 years was almost halved, suggesting again how HF disease modifying drugs can greatly affect the trajectory of the disease with an even more pronounced effect on HF prognosis [41].

### Limitations

This study was conducted before the introduction of SGLT2 inhibitors as a standard therapy for HFrEF, so it is not possible to estimate how well these results can be reproduced in a population also treated with these drugs. In addition, a chronic, stable and well-treated population from an outpatient setting, with normal right ventricular systolic function and capable of maximal exercise was included in the present study. Therefore, we do not know whether the same benefit can be expected in frailer patients or those in whom sacubitril/valsartan is introduced during acute or sub-acute HF events. It is impossible to rule out whether part of the observed functional improvement at CPET is also secondary to better adaptation toward exercise along the whole protocol (“training” effect). However, this can be considered minimal since these stable patients were enrolled among the ones already undergoing regular clinical and CPET follow-up at HF units and therefore used to regular exercise protocols. Lastly, it was not possible to run, albeit desirable, a placebo control study for ethical reasons, being sacubitril/valsartan treatment strongly recommended by ESC HF guidelines (class I) [42], as well as buy other guidelines [43, 44].

## Conclusion

Our study confirmed the positive effect of sacubitril/valsartan on functional capacity. For the first time, with a fixed and progressive monthly-based uptitration, we demonstrated how the more relevant CPET parameters, as well as echocardiographic measurements, biomarkers, NYHA class, and QoL, start to improve early, maintaining a progressive and sustained amelioration during the treatment. These results suggest a significant prognostic improvement as evidenced by the halving of the risk calculated with MECKI score.

## Data Availability

Raw data will be available upon request on the website www.zenodo.org.

## Declarations

### Disclaimer

All authors take responsibility for all aspects of the reliability and freedom from bias of the data presented and their discussed interpretation.

## References

[1] Conrad N, Judge A, Tran J, Mohseni H, Hedgecott D, Crespillo AP (2018). Temporal trends and patterns in heart failure incidence: a population-based study of 4 million individuals. Lancet.

[2] Savarese G, Lund LH (2017). Global public health burden of heart failure. Card Fail Rev.

[3] Tsao CW, Lyass A, Enserro D, Larson MG, Ho JE, Kizer JR (2018). Temporal trends in the incidence of and mortality associated with heart failure with preserved and reduced ejection fraction. JACC Heart Fail.

[4] McMurray JJ, Packer M, Desai AS, Gong J, Lefkowitz MP, Rizkala AR (2014). Angiotensin-neprilysin inhibition versus enalapril in heart failure. N Engl J Med.

[5] Solomon SD, McMurray JJV, Anand IS, Ge J, Lam CSP, Maggioni AP (2019). Angiotensin-neprilysin inhibition in heart failure with preserved ejection fraction. N Engl J Med.

[6] Velazquez EJ, Morrow DA, DeVore AD, Duffy CI, Ambrosy AP, McCague K (2019). Angiotensin-neprilysin inhibition in acute decompensated heart failure. N Engl J Med.

[7] Vitale G, Romano G, Di Franco A, Caccamo G, Nugara C, Ajello L et al (2019) Early effects of sacubitril/valsartan on exercise tolerance in patients with heart failure with reduced ejection fraction. J Clin Med 8 10.3390/jcm8020262 PMC6406731 30791533

[8] Goncalves AV, Pereira-da-Silva T, Galrinho A, Rio P, Soares R, Feliciano J (2020). Maximal Oxygen Uptake and Ventilation Improvement Following Sacubitril-Valsartan Therapy. Arq Bras Cardiol.

[9] Giallauria F, Vitale G, Pacileo M, Di Lorenzo A, Oliviero A, Passaro F et al. (2020) Sacubitril/valsartan improves autonomic function and cardiopulmonary parameters in patients with heart failure with reduced ejection fraction. J Clin Med 9 10.3390/jcm9061897 PMC7356720 32560431

[10] Malfatto G, Ravaro S, Caravita S, Baratto C, Sorropago A, Giglio A (2020). Improvement of functional capacity in sacubitril-valsartan treated patients assessed by cardiopulmonary exercise test. Acta Cardiol.

[11] Mapelli M, Vignati C, Paolillo S, De Martino F, Righini F, Agostoni P (2019). Sacubitril/valsartan can improve exercise performance in systolic chronic heart failure patients: a case report. Curr Med Res Opin.

[12] Sgorbini L, Rossetti A, Galati A (2017). Sacubitril/valsartan: effect on walking test and physical capability. Cardiology.

[13] Beltran P, Palau P, Dominguez E, Faraudo M, Nunez E, Guri O (2018). Sacubitril/valsartan and short-term changes in the 6-minute walk test: a pilot study. Int J Cardiol.

[14] Halle M, Schobel C, Winzer EB, Bernhardt P, Mueller S, Sieder C (2021). A randomized clinical trial on the short-term effects of 12-week sacubitril/valsartan vs. enalapril on peak oxygen consumption in patients with heart failure with reduced ejection fraction: results from the ACTIVITY-HF study. Eur J Heart Fail.

[15] Dos Santos MR, Alves MNN, Jordao CP, Pinto CEN, Correa KTS, de Souza FR (2021). Sacubitril/valsartan versus enalapril on exercise capacity in patients with heart failure with reduced ejection fraction: A randomized, double-blind, active-controlled study. Am Heart J.

[16] Piepoli MF, Hussain RI, Comin-Colet J, Dosantos R, Ferber P, Jaarsma T (2021). OUTSTEP-HF: randomised controlled trial comparing short-term effects of sacubitril/valsartan versus enalapril on daily physical activity in patients with chronic heart failure with reduced ejection fraction. Eur J Heart Fail.

[17] Lau CW, Martens P, Lambeets S, Dupont M, Mullens W (2019). Effects of sacubitril/valsartan on functional status and exercise capacity in real-world patients. Acta Cardiol.

[18] Campanile A, Visco V, De Carlo S, Ferruzzi GJ, Mancusi C, Izzo C et al (2023) Sacubitril/valsartan vs. standard medical therapy on exercise capacity in HFrEF patients. Life (Basel) 13 10.3390/life13051174 PMC10220971 37240819

[19] Bunsawat K, Ratchford SM, Alpenglow JK, Park SH, Jarrett CL, Stehlik J (1985). Sacubitril-valsartan improves conduit vessel function and functional capacity and reduces inflammation in heart failure with reduced ejection fraction. J Appl Physiol.

[20] Agostoni P, Dumitrescu D (2019). How to perform and report a cardiopulmonary exercise test in patients with chronic heart failure. Int J Cardiol.

[21] Guazzi M, Arena R, Halle M, Piepoli MF, Myers J, Lavie CJ (2016). 2016 Focused update: clinical recommendations for cardiopulmonary exercise testing data assessment in specific patient populations. Circulation.

[22] Ponikowski P, Voors AA, Anker SD, Bueno H, Cleland JGF, Coats AJS (2016). 2016 ESC Guidelines for the diagnosis and treatment of acute and chronic heart failure. Rev Esp Cardiol (Engl Ed).

[23] Agostoni P, Bianchi M, Moraschi A, Palermo P, Cattadori G, La Gioia R (2005). Work-rate affects cardiopulmonary exercise test results in heart failure. Eur J Heart Fail.

[24] Beaver WL, Wasserman K, Whipp BJ (1985). A new method for detecting anaerobic threshold by gas exchange. J Appl Physiol.

[25] Salvioni E, Mapelli M, Bonomi A, Magri D, Piepoli M, Frigerio M (2022). Pick your threshold: a comparison among different methods of anaerobic threshold evaluation in heart failure prognostic assessment. Chest.

[26] Wasserman K, Hansen JE, Sue DY, Stringer WW, Whipp BJ (2005). Clinical exercise testing principles of exercise testing and interpretation including pathophysiology and clinical applications. Lippincott Williams & Wilkins.

[27] Lang RM, Badano LP, Mor-Avi V, Afilalo J, Armstrong A, Ernande L (2015). Recommendations for cardiac chamber quantification by echocardiography in adults: an update from the American Society of Echocardiography and the European Association of Cardiovascular Imaging. Eur Heart J Cardiovasc Imaging.

[28] Spertus JA, Jones PG (2015). Development and validation of a short version of the Kansas city cardiomyopathy questionnaire. Circ Cardiovasc Qual Outcomes.

[29] Stubblefield WB, Jenkins CA, Liu D, Storrow AB, Spertus JA, Pang PS (2021). Improvement in Kansas city cardiomyopathy questionnaire scores after a self-care intervention in patients with acute heart failure discharged from the emergency department. Circ Cardiovasc Qual Outcomes.

[30] Agostoni P, Corra U, Cattadori G, Veglia F, La Gioia R, Scardovi AB (2013). Metabolic exercise test data combined with cardiac and kidney indexes, the MECKI score: a multiparametric approach to heart failure prognosis. Int J Cardiol.

[31] Agostoni P, Corra U, Cattadori G, Veglia F, Battaia E, La Gioia R (2013). Prognostic value of indeterminable anaerobic threshold in heart failure. Circ Heart Fail.

[32] Cacciatore F, Amarelli C, Maiello C, Mattucci I, Salerno G, Di Maio M (2020). Sacubitril/valsartan in patients listed for heart transplantation: effect on physical frailty. ESC Heart Fail.

[33] Nugara C, Giallauria F, Vitale G, Sarullo S, Gentile G, Clemenza F (2023). Effects of sacubitril/valsartan on exercise capacity in patients with heart failure with reduced ejection fraction and the role of percentage of delayed enhancement measured by cardiac magnetic resonance in predicting therapeutic response. Card Fail Rev.

[34] Corrado E, Dattilo G, Coppola G, Morabito C, Bonni E, Zappia L (2022). Low- vs high-dose ARNI effects on clinical status, exercise performance and cardiac function in real-life HFrEF patients. Eur J Clin Pharmacol.

[35] Mapelli M, Mattavelli I, Salvioni E, Banfi C, Ghilardi S, De Martino F (2022). Impact of Sacubitril/valsartan on surfactant binding proteins, central sleep apneas, lung function tests and heart failure biomarkers: hemodynamic or pleiotropism?. Front Cardiovasc Med.

[36] Mantegazza V, Volpato V, Mapelli M, Sassi V, Salvioni E, Mattavelli I et al (2021) Cardiac reverse remodelling by 2D and 3D echocardiography in heart failure patients treated with sacubitril/valsartan. Diagnostics (Basel) 11 10.3390/diagnostics11101845 PMC8534459 34679543

[37] Heidenreich PA, Bozkurt B, Aguilar D, Allen LA, Byun JJ, Colvin MM (2022). 2022 AHA/ACC/HFSA guideline for the management of heart failure: a report of the American College of Cardiology/American Heart Association Joint Committee on Clinical Practice Guidelines. Circulation.

[38] McDonagh TA, Metra M, Adamo M, Gardner RS, Baumbach A, Bohm M (2021). 2021 ESC Guidelines for the diagnosis and treatment of acute and chronic heart failure. Eur Heart J.

[39] Pastore MC, Mandoli GE, Giannoni A, Benfari G, Dini FL, Pugliese NR et al (2022) Sacubitril/valsartan reduces indications for arrhythmic primary prevention in heart failure with reduced ejection fraction: insights from DISCOVER-ARNI, a multicenter Italian register. Eur Heart J Open 2:oeab046 10.1093/ehjopen/oeab046 PMC9242049 35919657

[40] Johansson I, Joseph P, Balasubramanian K, McMurray JJV, Lund LH, Ezekowitz JA (2021). Health-related quality of life and mortality in heart failure: the global congestive heart failure Study of 23 000 patients from 40 countries. Circulation.

[41] Pezzuto B, Piepoli M, Galotta A, Sciomer S, Zaffalon D, Filomena D (2023). The importance of re-evaluating the risk score in heart failure patients: an analysis from the metabolic exercise cardiac kidney indexes (MECKI) score database. Int J Cardiol.

[42] Ponikowski P, Voors AA, Anker SD, Bueno H, Cleland JGF, Coats AJS (2016). 2016 ESC Guidelines for the diagnosis and treatment of acute and chronic heart failure: the task force for the diagnosis and treatment of acute and chronic heart failure of the European Society of Cardiology (ESC) developed with the special contribution of the Heart Failure Association (HFA) of the ESC. Eur Heart J.

[43] Ezekowitz JA, O'Meara E, McDonald MA, Abrams H, Chan M, Ducharme A (2017). 2017 Comprehensive update of the Canadian Cardiovascular Society Guidelines for the Management of Heart Failure. Can J Cardiol.

[44] Yancy CW, Jessup M, Bozkurt B, Butler J, Casey DE, Colvin MM (2017). 2017 ACC/AHA/HFSA focused update of the 2013 ACCF/AHA guideline for the management of heart failure: a report of the American College of Cardiology/American Heart Association Task Force on Clinical Practice Guidelines and the Heart Failure Society of America. J Am Coll Cardiol.

